# COVID-19 disease treatment: pivotal challenges in the arena of umbilical cord-mesenchymal stem cells (UC-MSCs)

**DOI:** 10.3389/fcell.2023.1146835

**Published:** 2023-05-19

**Authors:** Al-Hassan Soliman, Mohamed Abdellatif

**Affiliations:** ^1^ Faculty of Dentistry, Sinai University (SU), Arish, Egypt; ^2^ Faculty of Medicine, Zagazig University, Zagazig, Egypt

**Keywords:** COVID-19, stem cells, UC-MSC, cytokine storm, ARDS

## Abstract

This century’s first major epidemic of a new coronavirus illness (2019-nCoV) was a tremendous shock to the healthcare system. The onset of the pandemic has caused severe economic and health shortages. At this time, there are no viable treatments for COVID-19. Several clinical studies using cell-based therapies, such as umbilical cord mesenchymal stem cells, have showed promising results (UC-MSCs). UC-MSCs have been the focus of much study because to their potential as a treatment option for COVID-19 patients. Cytokine release syndrome, often called cytokine storm, increases the risk of morbidity and mortality from COVID-19. It has been established that UC-MSCs may suppress and control both the adaptive and innate immune responses by modulating the release of immunostimulatory cytokines. The purpose of this study is to assess and clarify the use of UC-MSCs for the treatment of ARDS caused by COVID-19.

## 1 Introduction

**FIGURE 1:**
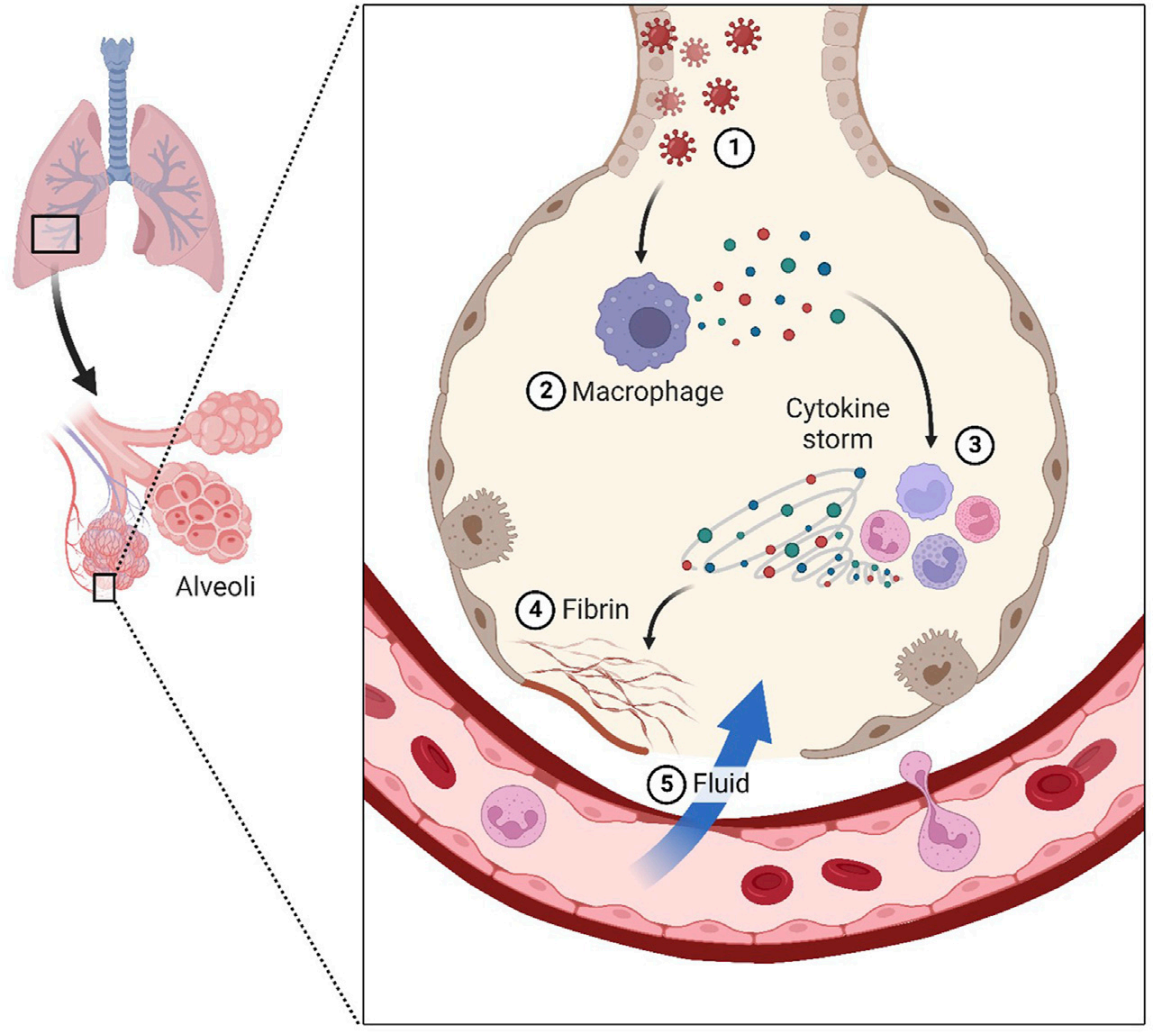
Coronavirus infects lung cells called pneumocytes (1). Immune cells, such as macrophages, recognize the virus and produce cytokines (2). Cytokines attract more immune cells, such as white blood cells, which produce more cytokines, resulting in an inflammatory cycle that damages lung cells “cytokine storm” (3). Additionally, the formation of fibrin can cause damage (4). Weakening blood vessels allow fluid to enter and fill the lungs, resulting in respiratory failure (5).

Increased fatalities and admissions to critical care units (ICUs) owing to respiratory infections, such as SARS coronavirus, have been linked to the 2019-nCoV infection. This severe acute respiratory syndrome outbreak has been linked to the type 2 coronavirus (SARS-CoV-2) (Xu et al., 2021). The severe acute respiratory syndrome coronavirus (SARS-CoV) and the Middle East respiratory syndrome coronavirus (MERS-CoV) are two novel and very fatal coronaviruses that have arisen in recent years. The lack of effective treatments for illnesses caused by 2019-nCoV may be attributed to the virus’s rarity. Most cases of ARDS in COVID-19 may be traced back to an inflammatory response known as a “cytokine storm.” However, the actual source of this remains unknown (Adas et al., 2021). These viruses, which are huge enveloped non-segmented RNA viruses, were initially identified in December 2019 in Wuhan, China (Adas et al., 2021). The incubation period of COVID-19 is typically 3–7 days but may go up to 14 days. Moderate to severe influenza-like illness, respiratory failure, and sepsis with multi-organ failure are all potential respiratory and non-respiratory symptoms. Warning symptoms include a high temperature, dry cough, and chest pain (Xu et al., 2021). The patient may have clinical signs such as a high body temperature, dry cough, difficulty breathing, muscle aches, and fatigue. (Adas et al., 2021). The most frequent reason for intensive care unit (ICU) admission is acute respiratory distress syndrome (ARDS) (Huang et al., 2020). Significant alveolar damage and basement membrane leaks characterize the devastating lung illness known as respiratory distress syndrome (ARDS) (Figure 1). Cell-based therapies using umbilical cord-mesenchymal stem cells are only one of several potential treatments for ARDS (UC-MSCs). Repair and regeneration of endothelium and alveolar cells, as well as targeted modulation of highly inflammatory immunological responses, are crucial for the recovery of ARDS patients. Coronavirus infection has no effective antiviral therapy. The disease’s genesis, epidemiology, length of human transmission, and clinical spectrum need to be further understood in the future.

## 2 The effect of COVID-19 on the CNS (brain structure and function)

While SARS-CoV-2 is often seen as a respiratory illness, it has been shown to cause damage to other organs, including the brain system (Mao et al., 2020).

These findings illuminate the systemic physiological effect of COVID-19, and being aware of these extrapulmonary aspects may aid in informing the overall prognosis in individuals who are afflicted. A wide variety of neurological symptoms, including those linked to the virus itself, the immune response, critical illness, associated therapy, and recovery, have been attributed to COVID-19 infection (Aghagoli et al., 2021).

Of 214 COVID-19 patients in Wuhan, China, 36.4% had neurologic signs, including central nervous system symptoms (24.8%), peripheral nervous system symptoms (8.9%), and skeletal muscle injuries (10.3%). Vertigo (16.8%) and headache (13.1%) were the most prevalent complaints in this group. Patients with more severe illnesses were more likely to have neurologic symptoms than patients with less severe disease, such as cerebrovascular disease (5.7% vs. 0.8%), altered consciousness (14.8% vs. 2.4%), and skeletal muscle damage (19.3% vs. 4.4%), than those with less severe illnesses (45.5% vs. 30.2%, respectively) (Mao et al., 2020).

Even though, there have been reports of probable cases of encephalitis caused by COVID-19. There was a single report of SARS-CoV-2 being discovered in the cerebrospinal fluid of a patient with symptoms. RT-PCR found SARS-CoV-2 RNA in the cerebrospinal fluid (CSF) of a 24-year-old Japanese male, but not in a nasal sample (Moriguchi et al., 2020). SARS coronavirus type 2 encephalitis was confirmed in this case (Moriguchi et al., 2020).

Another patient diagnosed with COVID-19 who was hospitalized had symptoms of meningeal irritation and altered state of consciousness. Both the CT scan of the brain and the lumbar puncture came out negative. Studies of the cerebrospinal fluid (CSF) for bacteria and viruses, including SARS-CoV-2 PCR testing, came back negative, but the patient was still diagnosed with COVID-19-associated meningoencephalitis, with the authors hypothesizing transient dissemination of the virus in the CSF with a strong inflammatory response (Ye et al., 2020).

Despite negative CSF PCR testing, the virus was found in frontal lobe neurons by electron microscopy during a *postmortem* study of a patient infected with SARS-CoV-2 who presented with disorientation and mental status abnormalities. This is the first direct evidence of SARS-CoV-2 in human brain tissue and suggests a possible direct hematogenous pathway for CNS seeding. Furthermore, viral particles were discovered in brain capillary endothelial cell and shown actively emerging from endothelial cells (Paniz-Mondolfi et al., 2020).

The cognitive damage seems to last long after COVID-19 has passed. In the 12 months following SARS-CoV-2 infection, there is an increased risk of cognitive and memory disorders (hazard ratio 1.77), and a diagnosis of Alzheimer Disease (hazard ratio 2.03), as seen in an analysis of the US Veterans Affairs national healthcare database (154,068 individuals diagnosed with COVID-19 from March 2020 to January 2021) (Xu et al., 2022).

SARS-CoV-2 infection has also been linked to acute demyelinating polyneuropathy. A 61-year-old asymptomatic Chinese lady arrived with sudden, widespread weakness and areflexia in her legs. SARS-CoV-2 infection was verified 8 days after the patient’s initial diagnosis of Guillain-Barré syndrome; the patient then exhibited classic COVID-19 symptoms. Both respiratory and neurological problems improved to the point that hospital release was possible (Zhao, Shen, Zhou, Liu, Chen).

## 3 Mesenchymal stem cells (MSCs): multipotential weapon

**FIGURE 2:**
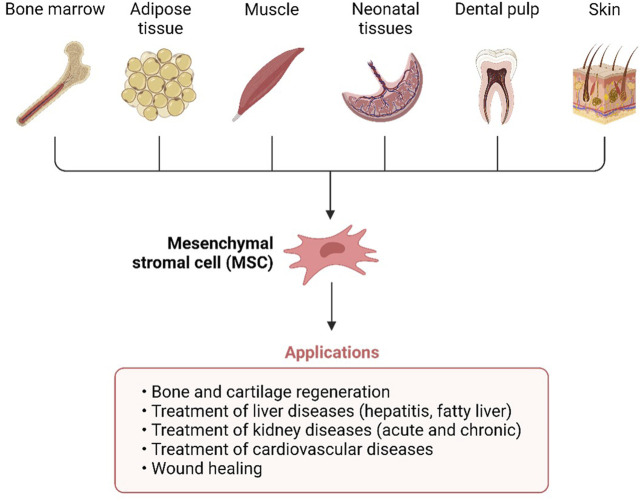
Umbilical cords (UC), fat, muscle, bone marrow, teeth, and skin are all good places to find mesenchymal stem cells.

Mesenchymal stem cells (MSCs) are non-hematopoietic cells having multi-lineage differentiation potential. They may be harvested from a variety of sources including bone marrow (BM), adipose tissue (fat), placental tissue (placenta), and the umbilical cord (UC) (Figure 2). Numerous mesenchymal stem cell (MSC) subtypes with potential medicinal applications are expressed in the human placenta (Wu et al., 2018). Wu, Mingjun, and others demonstrated that the human placenta is an MSC bank in addition to its critical functions in embryonic development, feeding, and tolerance. Furthermore, umbilical cord MSCs do not raise any ethical problems in contrast to embryonic stem cells (ESCs) (Wu et al., 2018). There is substantial evidence that UC-MSCs may promote tissue regeneration and repair. Additionally, their low immunogenicity is a result of their absence of surface MHC Class II expression, which makes them a desirable candidate for allogeneic transplantation and cell-based therapy (Lee and Song, 2018; Adas et al., 2021; Dilogo et al., 2021). Immune responses, both adaptive and innate, may be modulated by UC-MSCs thanks to their interesting immunomodulatory properties (Lee and Song, 2018; Adas et al., 2021; Dilogo et al., 2021). A focus on T lymphocytes, B lymphocytes, APCs, dendritic cells, and NK cells allows for regulation of adaptive immune cell activity, hence preventing non-autologous graft rejection (Lee and Song, 2018; Adas et al., 2021; Dilogo et al., 2021). Dendritic cells, NK cells, innate T helper (TH) cells, neutrophils, monocytes, macrophages, and mast cells are all potential targets for manipulating innate immunity. Similar to UC-MSCs, which have been demonstrated in several *in vitro* and animal studies to aid in wound healing and lessen the severity of infections, UC-MSCs have also been shown to aid in the regenerative processes (Lee and Song, 2018; Adas et al., 2021; Dilogo et al., 2021).

A breakthrough in the therapeutic strategy for COVID-19 therapy is particularly vital for the treatment of critically ill patients who may develop ARDS or dyspnea due to the lack of authorized or effective vaccines against COVID-19 infections (Lee and Song, 2018; Adas et al., 2021; Dilogo et al., 2021). So far, several clinical studies including remdesivir and dexamethasone have had encouraging outcomes. Excitingly, numerous labs have reported success with COVID-19 vaccines that have adequate safety, tolerability, and immunogenicity in early human clinical studies (Adas et al., 2021; Dilogo et al., 2021). The objective for this research was to see how well UC-MSCs responded against COVID-19-induced ARDS. Health has improved in many clinical studies using this particular kind of stem cell in individuals with COVID-19 (Table 1). And also the stem cell therapy, which has the ability to stop the cytokine storm caused by COVID-19 infection, is being investigated as a possible treatment for ARDS.

**TABLE 1:** Shows different study designs that used UC-MSCs as adjuvant therapy for COVID-19.

Number of patients	References	Study design	Sample size		Date	Stem cell type
			Experiment group	Control group		
11	Hashemian et al. (2021)	Phase 1 clinical trial	-	-	15/03 to 10/04 2020	MSCs (UC/PL-MSC)
41	Huang et al. (2020)	Cohort study	-	-	16/12 2019, to 02/01 2020	-
40	Dilogo et al. (2021)	Randomized controlled trial	20	20	-	(UC-MSCs)
100	Shi et al. (2021)	Randomized phase 2 trial	65	35	05/03 to 28/03 2020	(UC-MSCs)
30	Adas et al. (2021)	Clinical trial	10	20	-	WJ-MSCs
44	Xu et al. (2021)	Nonrandomized controlled trial	26	18	01 to 04/2020	UC-MSC
18	Meng et al. (2020)	Controlled non-randomized	9	9	-	UC-MSCs
41	Shu et al. (2020)	Randomized controlled trial	12	29	12/02 to 25/03/2020	UC-MSCs
24	Lanzoni et al. (2021)	Randomized controlled trial	12	12	25/04 to 21/07/2020	UC-MSCs

## 4 Umbilical cord mesenchymal stem cells in brain homeostasis

UC-MSCs have many advantages compared with other cell types, such as the minimum risk of allograft rejection (Wang et al., 2013). When transplanted, Wand and coworkers’ study aimed to certify the compensation of neurological recovery provided by the migration and differentiation of UC-MSCs or the neurotrophic factors (Wang et al., 2013). Increasing evidence demonstrates the capability of UC-MSCs to exert a protective effect after injury in different organs through paracrine production of mitogenic, antiapoptotic, and trophic factors through their immunomodulatory action and by their ability to efficiently scavenge reactive oxygen species (ROS) (Valle-Prieto and Conget, 2010). Currently, increasing literature has revealed that UC-MSCs transplantation significantly restored brain functions in patients with sequelae of traumatic brain injury (Wang et al., 2013). These cells display strong self-renewal and differentiation abilities (Cui et al., 2011). Hence, they could improve neurological function (Seo et al., 2011). When induced by chemical and neurotrophic factors, UC-MSCs can differentiate into vascular endothelial cells, neural cells, and glial cells with secretory functions important for brain functions and homeostasis (Figure 4) (Koh et al., 2008).

**FIGURE 3:**
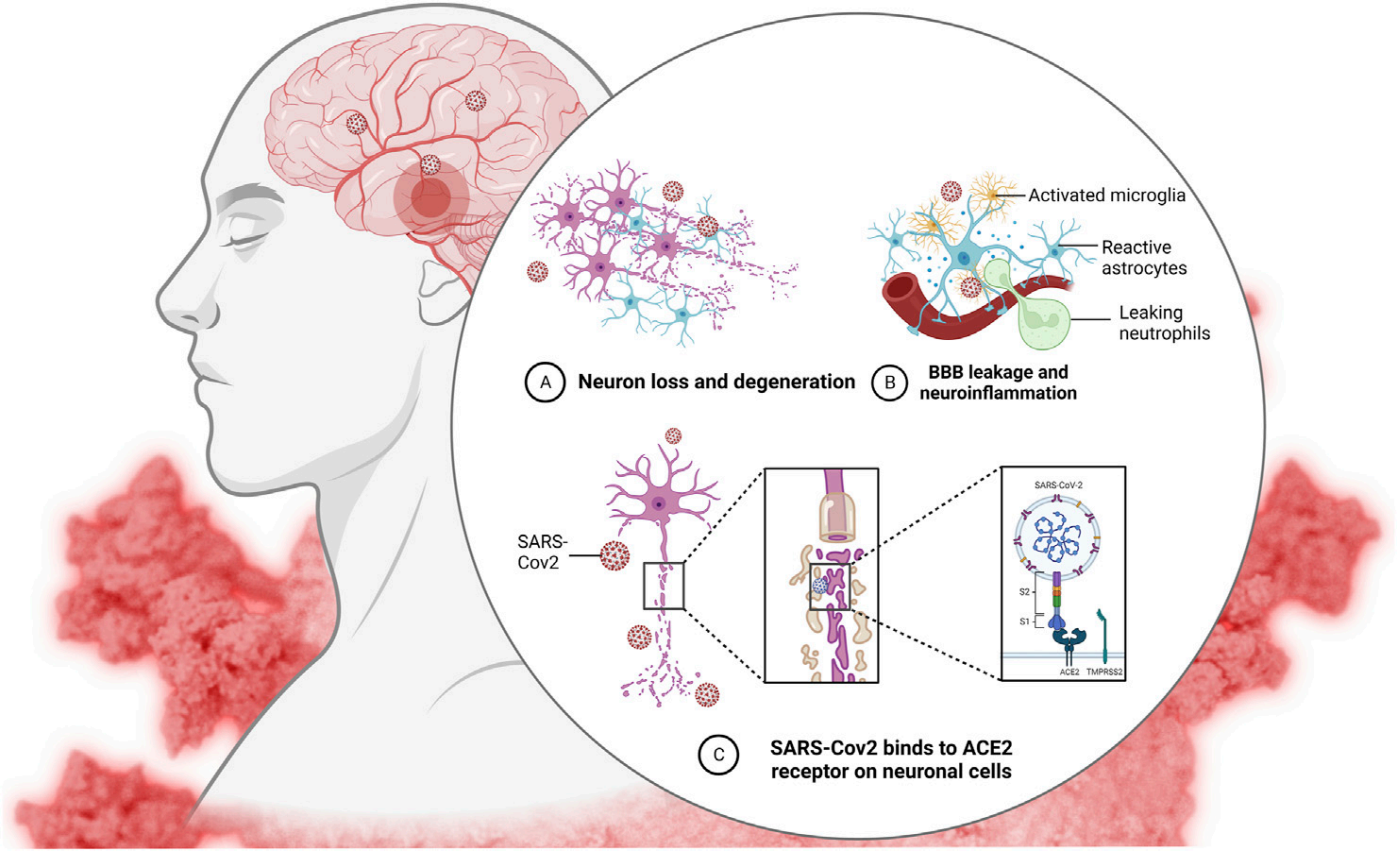
Effect of cytokine storm on the brain and CNS.

**FIGURE 4:**
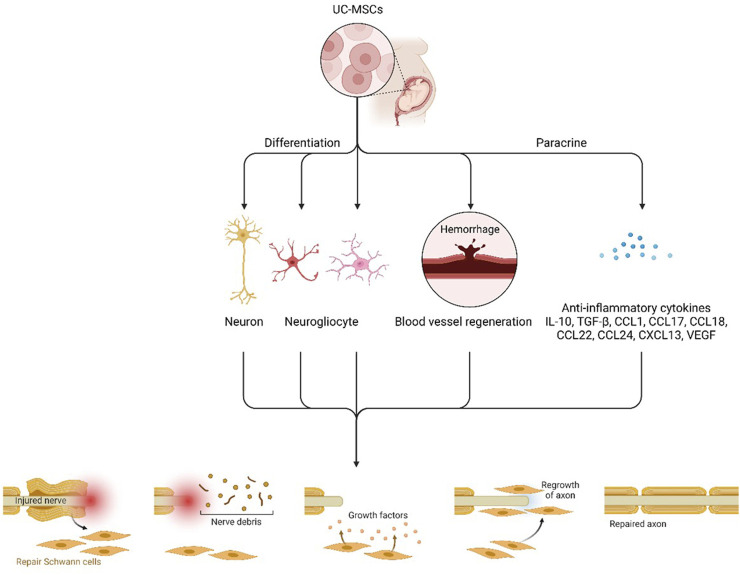
Hypothetical scheme of the therapeutic mechanisms of UCB-SCs. UCB-SCs might proliferate, migrate, and differentiate into “replacement” glial cells and neurons and subsequently integrate into and play a functional role in the host brain. UCB-SCs might also enhance blood vessel regeneration and act as transporters to deliver restorative and neuroprotective factors and endogenous cell signals to injured host neural cells in a paracrine manner immune factors and anti-inflammatory cytokines.

Researchers demonstrated the paracrine potential of UC-MSCs in promoting neural renewal, neural protection and preventing inflammation at the site of spinal cord injury, with evidence showing it to be additionally able to further restore brain homeostasis and physiological equilibrium (Han et al., 2022). UC- MSCs have shown reduced expression of IL-1β and increased expression of neural growth factor (NGF) in the treated spinal cord tissue; this results in the renewal of motor function and integrity of the spinal cord (Chudickova et al., 2019). Also, Kim et al. have shown recovery of the behavioral role in hypoxic-ischemic encephalopathy (HIE)-induced brain infarcted rats via anti-astroglia, anti-apoptotic, and anti-inflammatory factors of UC- MSCs (Ahn et al., 2021). In another study, Son et al. examined whether sRAGE-secreting UC-MSCs via CRISPR/Cas9 technique protect neuronal cell death in a Parkinson’s Disease animal model (Son et al., 2017). The study revealed that decreasing AGE-RAGE binding may be the potential therapeutic approach for curing Parkinson’s Disease by preventing neuronal cell death and achieving brain health and homeostasis (Chetty et al., 2022).

Seo and others investigated whether hUCB-MSCs could differentiate into neuronal cells and how hUCB-MSCs could protect against neuronal cell death (Seo et al., 2011). Consistent with the effect of BM-MSCs, researchers demonstrated the dynamic role of hUCB-MSCs, via activation of the PI3K/AKT and JAK2 STAT3 signaling pathways, and their differentiation into functional neurons in the cerebrum, which promoted Purkinje cell survival in the cerebellum (Seo et al., 2011). Hence, increasing brain function and homeostasis levels. Recently, the authors found that the transcription factor REX1 regulates the proliferation/differentiation of human MSCs through the suppression of p38 mitogen-activated protein kinase (MAPK) signaling via the direct suppression of mitogen-activated protein kinase 3 (MKK3) (Seo et al., 2011). Moreover, decreased histone deacetylase (HDAC) activity is important for MSC self-renewal by balancing polycomb group genes and the jumonji domain containing 3 expressions. These findings suggest that transcriptional regulation and chromatin modification might define the cellular microenvironment in which a deficiency of NPC1 leads to disease endpoints (Seo et al., 2011).

In the future, UC-MSCs will be investigated for their ability to treat spinal cord injuries, stroke sequelae, cerebral palsy, and other neurological diseases to restore brain homeostasis and physiological functions. Our next step is to prepare collaborations with imaging centers and neuro-electrophysiological and urodynamics centers to perform large sample-size clinical trials to define UC-MSCs as a promising candidate for the treatment of all diseases of the CNS (Chetty et al., 2022).

## 5 UC-MSCs applications in COVID-19 treatment

UC-MSCs were shown to considerably reduce or eliminate clinical symptoms including chest tightness, shortness of breath, and weariness, according to study by Shu et al. Depending on the severity of the damage, lymphocyte counts and other inflammatory indicators may rebound to pre-injury levels. Arterial blood gas levels showed that patients in the experimental group recovered from chest tightness and shortness of breath more quickly than those in the control group (Shu et al., 2020). In the years between 2014 and the COVID-19 pandemic, around 30 clinical studies using MSCs to treat ARDS were published on ClinicalTrials.gov website (Harrell et al., 2019; Khoury et al., 2020). Tests such as a full blood count, coagulation profile, and serum biochemistry were used as benchmarks in clinical trials. Real-time polymerase chain reaction (RT-PCR) methods were used to identify SARS-CoV and MERS-CoV in respiratory specimens such nasal and throat swabs, bronchoalveolar lavage fluid, sputum, and bronchial aspirates (Huang et al., 2020). There was also routine testing for bacteria and yeasts. Patients were admitted with leucopenia and lymphopenia due to low white blood cell and lymphocyte counts (Huang et al., 2020). ICU patients also had a longer prothrombin time and higher D-dimer values. Eleven individuals with COVID-19-induced ARDS were reported by Hashemian et al., to have been hospitalized to critical care units (ICU) (Hashemian et al., 2021). Surprisingly, UC-MSC therapy dramatically accelerated the disappearance of lung solid component lesions as compared to a placebo (Shi et al., 2021). With these findings in mind, it is reasonable to think of including UC-MSC in the current treatment protocol for people with COVID-19. Conditional clearance for “extended access compassionate use” of MSCs in patients with COVID-19 was recently decided upon by the US Food and Drug Administration (FDA). Differentiation of T helper 2 (Th2) cells into Tregs, which suppress inflammation, is induced by these UC-MSCs (Dilogo et al., 2021). Treatment with UC-MSCs significantly decreased the “cytokine storm” of inflammatory cytokines caused by COVID-19 (Sánchez-Guijo et al., 2020; Shi et al., 2021). It has been established that MSCs may inhibit overactive immunological and inflammatory processes, stimulate tissue healing, and release antimicrobial compounds (Lanzoni et al., 2021).

## 6 UC-MSCs mechanism of action

Sánchez-Guijo and others used allogenic adipose tissue-derived MSCs and found a 15% mortality rate (2 out of 13 cases) (Sánchez-Guijo et al., 2020). Another study, on the other hand, reported a high mortality rate in their cases (5 out of 11) (Hashemian et al., 2021). Some of the issues that Hashemian et al., pointed out were subsequently blamed for this. In the research conducted by Sánchez-Guijo et al., for instance, the patient’s general health improved significantly. It was shown that having them in one’s life was associated with a lower probability of contracting other illnesses. In addition, their case series was only followed-up on for 16 days, but Hashemian et al. used a longer follow-up period (60 days to report an endpoint). UC-MSCs have been proposed as a therapy for COVID-19 patients with lung injury, but this is the first randomized, double-blind, placebo-controlled experiment to assess their safety and effectiveness (Hashemian et al., 2021). Lung solid component lesions were eliminated rapidly and safely after UC-MSC therapy, and the potential for integrated regeneration was enhanced. Lung damage caused by COVID-19 may be treated non-invasively using UC-MSCs. When MSCs are injected into the body, they may swiftly stimulate the host’s innate immune cascade system, which includes complement and blood coagulation, and so generate blood-mediated inflammatory responses (Xu et al., 2021).

## 7 Cytokine storm: Inflammation-inducing warrior

A cytokine storm is the most deadly cause of COVID-19 ARDS symptoms. The wide range of local and systemic symptoms seen with COVID-19 infection is most likely due to cytokine storms (Adas et al., 2021). Acute respiratory distress syndrome (ARDS), heart damage, subsequent infection, systemic inflammatory response syndrome (SIRS), and multisystem failure are all possible outcomes. Preventing a cytokine storm in COVID-19 infected patients might have a profound impact on their care. Several lines of evidence point to the fact that MSCs connect to active immune cells, clustering them together to increase their immunosuppressive effects. Various *in vivo* lung disease models have been used to examine MSCs. Clinical trials have indicated that mesenchymal stem cells (MSCs) used to treat influenza increase alveolar fluid evacuation and reduce lung damage. MSC transplantation has been suggested as a potential immune modulator for the initiation of an inflammatory cytokine storm. Mortality and morbidity from COVID-19 are exacerbated by a condition known as cytokine release syndrome (CRS), which is produced by a cascade of immune cells secreting pro-inflammatory cytokines. Blood levels of inflammatory cytokines are significantly elevated in CRS. There has been an increase in the inflammatory alveolar macrophage population while there has been a decrease in the secretion and recruitment of pro-inflammatory cytokines (Adas et al., 2021). Adas et al. aimed to reduce the severity of the cytokine storm, determine the efficacy of the therapy in promoting healing, and probe its underlying processes by administering UC-MSCs to very sick COVID-19 patients. In order to determine the treatment’s mechanism, researchers measured the levels of growth factors, apoptotic markers, chemokines, matrix metalloproteinases, and granzyme-B, as well as assessed lymphocyte subsets and overall oxidant/antioxidant status indicators. Patients suffering from severe cases of COVID-19 had noticeably elevated IL-6 levels. Growth factors (TGF-b, VEGF, KGF), anti-inflammatory cytokines (e.g., IL-10, IL-13, IL-1ra), and proinflammatory cytokines (e.g., IFNg, IL-6, IL-17A, IL-2, IL-12) were also assessed (Lanzoni et al., 2021). It is encouraging to see that a reduction in inflammatory cytokines is more strongly linked to the favorable response in UC-MSC therapy individuals. This study’s results have potential application to studies examining COVID-19, ARDS, inflammation, immunological response, and autoimmune (Lanzoni et al., 2021). Moreover, it increases the levels of several growth factors and modulates the chemokine pathway, both of which aid in the repair of damaged organs after infection (Adas et al., 2021).

## 8 UC-MSCs can reduce inflammatory responses

By inhibiting alveolar collapse, cell death, and collagen formation in lung tissues, MSCs obtained from umbilical cords have been found to be useful in the treatment of inflammatory lung disorders (Harrell et al., 2019). The ACE2 receptor is the primary entry receptor for SARS-CoV-2 (Figure 3) (Walls et al., 2020). Although MSCs do not express ACE2, investigations conducted *in vitro* have demonstrated that they may survive after being exposed to SARS-CoV-2 infected cells (Xu et al., 2021). Patients with severe COVID-19 may benefit from MSC therapy because it reduces pro-inflammatory cytokines, which promote immunological dysfunction and impair clinical outcomes in COVID-19 (Rogers et al., 2020). Serum IL-6 is one example of a cytokine that has been linked to COVID-19 disease development. Patients’ health dramatically improved in the MSC treatment group, as shown by a number of anti-inflammatories, anti-fibrosis, and immunomodulatory markers. The cytokine storm may be calmed, and disease progression slowed with conventional therapy based on mesenchymal stem cell transplantation. Organ damage was mitigated, and healing time was cut in half by MSC-mediated proliferation and differentiation. This research shows that MSCs have a favorable safety profile, may reduce mortality and ICU stay, and serve a specialized therapeutic function in the treatment of critically sick patients (Adas et al., 2021). When the microenvironment is enhanced with MSC treatment, the immune system is less likely to overreact, and the body’s natural ability to heal is able to take over. Mesenchymal stem cells (MSCs) have been proven to enhance lung microenvironment, protect alveolar epithelial cells, decrease pulmonary fibrosis, and boost lung function when administered intravenously (Shu et al., 2020).

## 9 Intravenous injection of UC-MSCs

Once MSCs are injected intravenously, the vast majority of these cells quickly find themselves ensnared in the lung’s capillary beds (Shi et al., 2021). Since the lungs are the principal site of damage in ARDS, transplanted UC-MSCs are most effective when administered intravenously because of the high number of cells that may be delivered at once (Leng et al., 2020; Shi et al., 2021). Patients with moderate to severe COVID-19 have benefited by the infusion of UC-MSCs through intravenous route, as has been established in several trials (Meng et al., 2020). When high-dose allogeneic UC-MSCs were infused repeatedly into a select group of critically sick patients with COVID-19-induced ARDS, they improved respiratory distress and reduced inflammatory biomarkers (Hashemian et al., 2021). The researcher opted for an IV infusion since it has been shown to successfully deliver a high cell concentration to the lungs in cell-based therapy for ARDS (Hashemian et al., 2021). The results of this experiment suggest that it is safe to provide UC-MSC infusions to patients with ARDS who have tested positive for the COVID-19 virus. Three of the nine patients who got UC-MSCs showed temporary unfavorable effects, according to the study by Meng et al. Two of them had temporary flushing of the face and fever, and one experienced temporary hypoxia 12 h after receiving UC-MSCs (Meng et al., 2020).

## 10 Conclusion and future perspectives

There is not yet a cure for COVID-19 (Khoury et al., 2020; Lanzoni et al., 2021). For this reason, MSC-based treatment may one day be used to treat COVID-19. Large, randomized, multi-center clinical research is needed to correctly assess the therapeutic potentials of MSC in COVID-19-induced ARDS. As reported by Leng et al., All seven patients with COVID-19 pneumonia in his study showed significant improvements in outcomes after receiving 1,106 cells/kg of MSCs from a commercial source, (Leng et al., 2020). In comparison to the control group, the UC-MSCs group had a survival rate that was 2.5 times greater, according to study by Dilogo et al. (2021). When patients with multiple diseases were treated with UC-MSCs, their survival rates increased relative to those in the control group by a factor of 4.5. According to the findings of Shu et al., hUC-MSC therapy is a very successful and promising noninvasive treatment for severe COVID-19 abnormalities (Shu et al., 2020). It is essential to conduct a phase three trial to determine whether or not UC-MSC treatment is effective in preventing long-term pulmonary damage, decreasing death, and elucidating underlying mechanisms in COVID-19 illness (Shi et al., 2021). Although hUC-MSCs have been shown to have a beneficial impact on severe COVID-19, the precise molecular mechanism by which this occurs is unclear and hence needs additional investigation (Shu et al., 2020). New evidence reveals that mesenchymal stem cells (MSCs) in lung disease, such as ARDS, may use paracrine chemicals and/or alternate methods of action, such as gap junctions, tunneling nanotubes, and extracellular vesicles, to govern cell death (Shu et al., 2020).
